# Application of Induced Pluripotent Stem Cells in Malignant Solid Tumors

**DOI:** 10.1007/s12015-023-10633-y

**Published:** 2023-09-27

**Authors:** Rong He, Zhijie Weng, Yunkun Liu, Bingzhi Li, Wenxuan Wang, Wanrong Meng, Bo Li, Longjiang Li

**Affiliations:** 1 State Key Laboratory of Oral Diseases, National Clinical Research Center for Oral Diseases, Department of Head and Neck Oncology, West China Hospital of Stomatology, Sichuan University, Chengdu, China; 2 State Key Laboratory of Oral Diseases, National Clinical Research Center for Oral Diseases, Department of Orthodontics, West China Hospital of Stomatology, Sichuan University, Chengdu, China

**Keywords:** Induced pluripotent stem cells, Cancer cell reprogramming, Malignant solid tumors, Cancer stem cell, Immunotherapy

## Abstract

In the past decade, induced pluripotent stem cells (iPSCs) technology has significantly progressed in studying malignant solid tumors. This technically feasible reprogramming techniques can reawaken sequestered dormant regions that regulate the fate of differentiated cells. Despite the evolving therapeutic modalities for malignant solid tumors, treatment outcomes have not been satisfactory. Recently, scientists attempted to apply induced pluripotent stem cell technology to cancer research, from modeling to treatment. Induced pluripotent stem cells derived from somatic cells, cancer cell lines, primary tumors, and individuals with an inherited propensity to develop cancer have shown great potential in cancer modeling, cell therapy, immunotherapy, and understanding tumor progression. This review summarizes the evolution of induced pluripotent stem cells technology and its applications in malignant solid tumor. Additionally, we discuss potential obstacles to induced pluripotent stem cell technology.

**Graphical Abstract:**

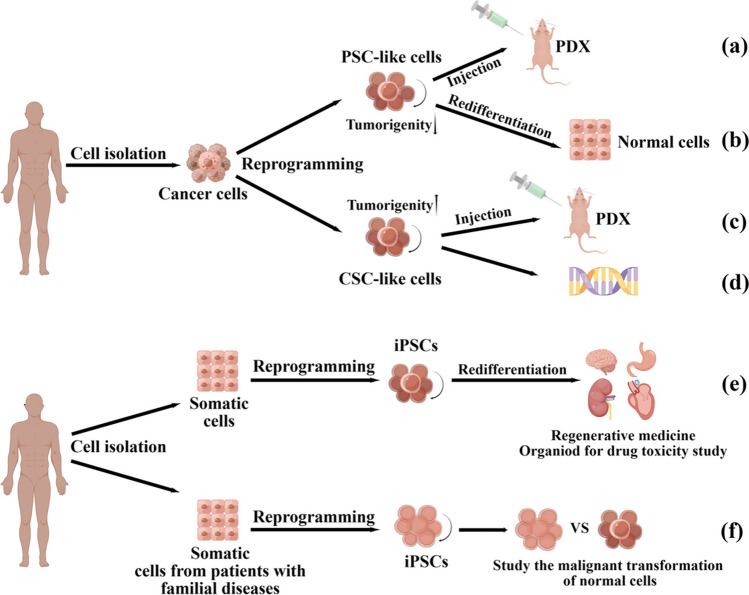

## Background

Malignant solid tumors are difficult to treat thoroughly with conventional treatments due to incomplete local resection, metastasis, invasion, drug resistance, and recurrence. Additionally, therapies, including drugs, radiotherapy, and chemotherapy have unpleasant side effects that exacerbate patients' discomfort [1]. Researchers have spent many years studying the development and pathogenesis of malignant solid tumors to improve treatments. Traditional models have been an essential tool in cancer research; however, they have limitations, including a lack of heterogeneity, low efficiency, and difficulty simulating cancer progression. The iPSCs technology developed in recent years is anticipated to enhance traditional models. Recent human embryonic stem cells (ESCs) applications have introduced a novel approach to cancer research [2]. However, ethical and safety concerns regarding ESCs have long impeded their applications, and the development of iPSCs technology appears to address these concerns. iPSCs have several advantages due to their human origin, ability to expand in culture, accessibility, and ability to differentiate into virtually any desired cell type. Additionally, the use of patient-specific iPSCs offers the possibility of personalized medicine. Therefore, iPSCs technology is advantageous for disease modeling and advancing the development of cancer treatment modalities [3].

## The Development and Applications of iPSCs Technology

**Fig. 1:**
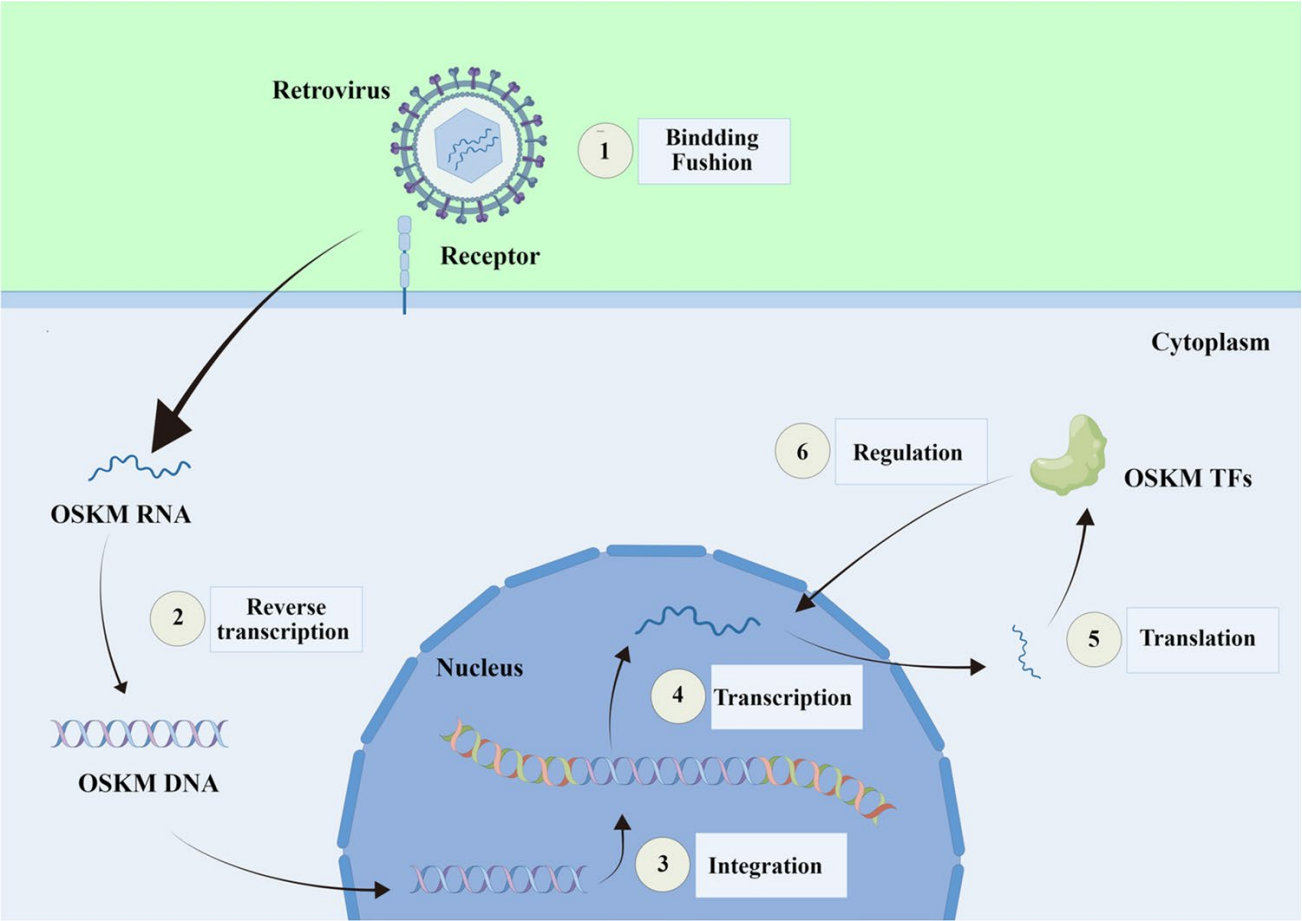
The classic process of ectopic expression of OSKM transcription factors. Retroviruses initiate cellular reprogramming by introducing RNA encoding OCT4, SOX2, KLF4, and c-Myc transcription factors into the cell. Subsequently, after reverse transcription, this genetic material binds to the cell's nuclear DNA

**Fig. 2:**
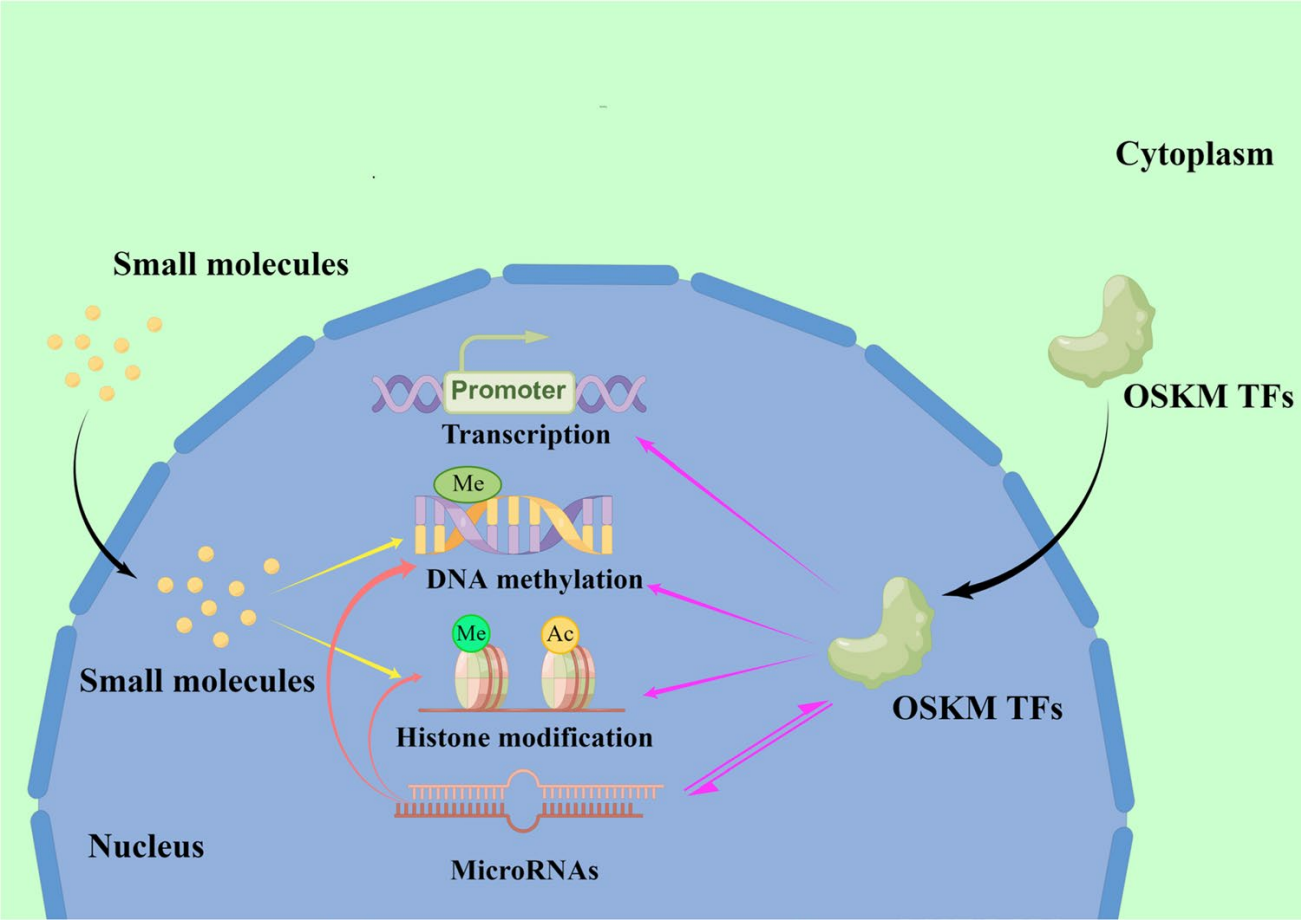
The mechanism of generating induced pluripotent stem cells. The OSKM transcription factors promote cell reprogramming via various methods, including binding to promoters, regulating epigenetics, and interacting with microRNAs. The epigenetic modifications include DNA methylation and histone modification. MicroRNAs can also promote cell reprogramming via epigenetic regulation. Small molecule compounds can improve reprogramming efficiency or reprogram cells independently

In 1958, Gurdon successfully produced healthy tadpoles by transferring the nuclei from intestinal epithelial cells to enucleated Xenopus laevis cells [4]. This breakthrough experiment established the basis for the field of cell reprogramming. In 2006, Shinya Yamanaka et al. [5] successfully transferred four specific genes (OCT4, SOX2, KLF4, and c-Myc; OSKM) into differentiated cells in mice that could reverse their differentiation process and produce cells with functional similarity to ESCs and were named iPSCs. Using the same technique, Yamanaka's group generated iPSCs from human fibroblasts a year later [6]. Since then, scientists have developed numerous techniques for generating iPSCs, including retroviral transduction [7], lentiviral transduction [8, 9], elastin-like polypeptide (ELP) based gene delivery [10], episomal plasmid [11, 12], Sendai virus [13, 14], modified mRNA [15, 16], transposons [17], and small-molecule compounds [18]. The methods used to generate iPSCs are summarized in Table 1. Small molecule compound-mediated reprogramming is the most promising of these methods due to its simplicity, safety, and effectiveness [19–22]. The ectopic expression of OSKM and the mechanism for generating iPSCs are described in Fig. 1, 2.

**Table 1:** Overview of the methods for generating iPSCs

	Methods	Cell type	Media	Safety	Efficiency (approximate)	References
Integrating methods	Retrovirus	Human fibroblasts	OSKM	( +)	0.01%	[6]
	Lentivirus	Human fibroblasts	OSLN	(+ +)	0.001% ~ 0.02%	[9]
Non integrating methods	Sendai virus	Human fibroblasts	OSKM	(+ + +)	1%	[14]
	Modified mRNAs	Human fibroblasts	OSKM	(+ + +)	4.40%	[16]
	Episomal plasmid	Mouse fibroblasts	OSKM	(+ + +)	0.003%	[12]
	Elastin-like polypeptide	Mouse fibroblasts	OSKM	(+ + +)	0.004% -0.007%	[10]
	Transposon	Rhesus macaque fibroblasts	OSKMLN	(+ + +)	0.08–0.12%	[17]
	Adenovirus	Mouse fibroblasts	OSKM	(+ + +)	< 0.0001%	[12]
	Recombinant proteins	Mouse fibroblasts	OSKM	(+ + +)	0.006%	[163]
	Single polycistronic vector	Mouse fibroblasts	OSKM	(+ + +)	0.0001%	[164]
	Chemical induction	Mouse fibroblasts	Small molecule compounds	(+ + + +)	> 50%	[21]

OSKM: OCT4, SOX2, KLF4, c-Myc; OSLN: OCT4, SOX2, Lin28, NANOG; OSKMLN: OCT4, SOX2, KLF4, c-Myc, Lin28, NANOG.

The applications of iPSCs technology to model disease, screen drugs, reverse the malignant phenotype of cancer cells, and develop new therapeutic modalities have produced significant results in the study of malignant solid tumors. Additionally, iPSCs technology has been extensively used in the study of neurodegenerative disorders [23–25], leukemia [26, 27], liver fibrosis [28], cardiovascular diseases [29, 30].

## Application of iPSCs in malignant solid tumor modeling

**Fig. 3:**
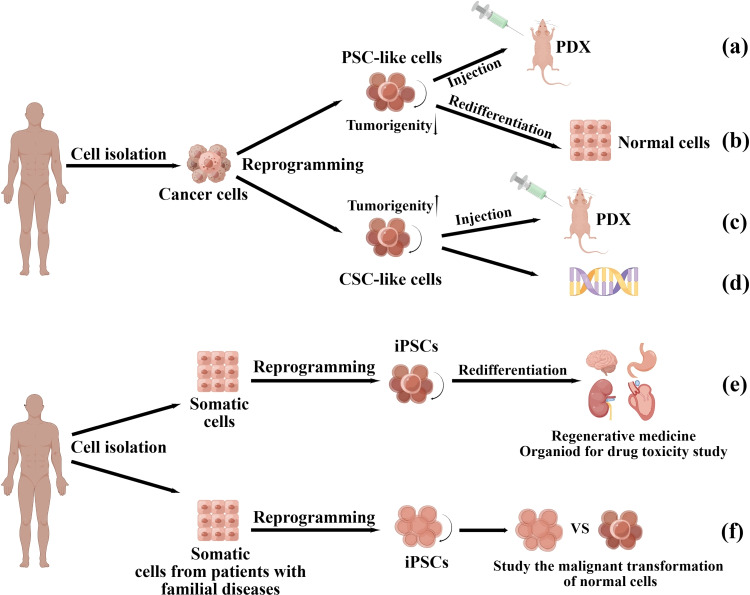
The applications of cancer cell and somatic cell reprogramming. There are two main outcomes of cancer cell reprogramming: PSC-like cells or CSC-like cells. PSC-like cells can be used to construct PDX (**a**). Re-differentiated PSC-like cells can become normal cells to further reduce tumorigenicity (**b**). CSC-like cells can be used to construct CSCs models and further study the characteristics of CSCs (**c**, **d**). Somatic cell reprogramming and redifferentiation can generate various cell types (**e**). Reprogrammed somatic cells from patients with family diseases can help to understand the malignant transformation of normal cells (**f**)

Identifying the underlying pathological mechanisms of tumors is crucial for developing novel therapeutic strategies. The use of cell lines for tumor modeling is limited due to their lack of heterogeneity. Tumor models based on patient-derived cells, such as patient-derived xenograft (PDX) and patient-derived organoid (PDO) models, are highly valuable in studying the etiology of tumors. However, the lack of expandable sources of primary cells, particularly for difficult-to-access cells such as brain and heart cells, limits their application. iPSCs derived from readily available cells can self-renew indefinitely and differentiate into all cell lineages of an organism, providing a powerful and unlimited source for generating differentiated cells (Fig. 3e). Additionally, iPSCs derived from individuals with an inherited predisposition to develop cancer may mimic the early stage of tumor development and help in understanding the progression of tumors. The potential of iPSCs in cancer modeling can help researchers better understand cancer development and progression.

## Application of iPSCs in PDXs

Patient-derived xenografts (PDXs) have emerged as a prominent model system that can accurately capture primary and metastatic malignancies' cellular, molecular, and physiological features [31]. In addition, PDXs are gaining attraction in fields including biomarker identification, drug development, and assessment of drug responses [32, 33]. However, establishing PDX models for primary tumors of some patients can be challenging, as many cannot be directly transplanted into immunodeficient mice. In these cases, reprogramming primary tumor cells into iPSCs can be beneficial [34]. This junction permits genetic manipulation of the cells in vitro before transplantation and can facilitates the tracking and study of their effects on tumor growth in vivo. Transplanting iPSCs or their derivatives into suitable animal models can increase their experimental value by creating a more physiological, three-dimensional, in vivo environment.

Pancreatic ductal adenocarcinoma (PDAC) was believed to have a dismal prognosis. Before the study by Zaret et al. [35], no dynamic, live human cellular models that underwent early developmental stages. Subcutaneous injection of iPSCs into immune-compromised animals is used to test the pluripotency of iPSCs by developing teratomas. Injected PDAC-iPSCs showed that growing teratomas produced ductal structures with a more pronounced architectural organization that resembled PanIN-stage-like structures compared to controls. Cellular and molecular analysis confirmed that these formations eventually advanced to the aggressive PDAC stage. The protocol for reprogramming PDAC cells into pluripotent stem cell-like lines was published in 2019 [36]. In 2013, a piggyBac transposon vector was used to reprogram glioblastoma-derived neural stem cells (GNS) using only two factors, OCT4 and KLF4 [37]. GNS-derived iPSCs show extensive resetting of cancer-specific methylation, and after neural differentiation, iPSCs are highly tumorigenic when injected into immunodeficient mice. The PDXs established for glioblastoma can be used to further investigate the characteristics of this tumor. Figure 3a, c partly described the applications of iPSCs in PDXs.

## Application of iPSCs in PDO

Organoids are three-dimensional cultures derived from primary cells that are structurally, functionally, and genetically similar to in vivo organs, and they can be used to bridge the gap between two-dimensional cultures and in vivo animal models [38]. Patient-derived tumor organoids can be grown directly from tumor tissues or through genetic modification of normal tissues, including CRISPR gene editing [39]. Even after prolonged culture, organoids retain their parental tumors' histological structure, genomic structure, and gene expression profile [40]. In 2009, Hans et al. [41] were able to cultivate small intestinal organoids from LGR5 + small intestinal stem cells. Since then, scientists have conducted multiple experiments and established many organoids derived from healthy tissues and long-term organoid cultures derived from the primary colon, esophagus, pancreas, stomach, liver, endometrium, and breast cancer tissues [42]. These organoids are extensively used in cancer research, including drug screening, toxicity testing, and basic cancer research.

Traditional organoid models are generated from primary tissue biopsies, so most expanded cells are adult stem cells (ASCs), even though they offer significant advantages over two-dimensional culturing [43]. Initially, ASCs were believed to have limited in vitro proliferative potential [44]. In contrast, iPSC-derived organoids have the advantage of not relying on primary tissue resection. Once a patient has established an iPSCs cell line, these cells can be replicated indefinitely to generate multiple cell types [45]. Liver [46, 47], lung [48], stomach [49], cardiomyocytes [49], pancreatic duct [50, 51], fallopian tube [52], intestine [53], and other organoids have been established by using iPSCs (iPSCs-derived organoids). iPSCs-derived organoids can generate more complex structures and vascularized models than conventional organoids, which lack the native microenvironment of stromal cells, muscle, blood vessels, and immune cells. One study successfully integrated stromal components, such as the vascular system, fibroblasts, and immune cells, using iPSC-induced mesodermal progenitors [43]. Additionally, Hideki Taniguchi et al. [54] have generated functional, three-dimensional sheet-like human hepatocellular carcinoma (HCC) organoids in vitro, using luciferase-expressing Huh7 cells, human iPSC-derived endothelial cells (iPSC-EC) and human iPSC-derived mesenchymal cells(iPSC-MC). A mouse model of HCC was successfully generated by transplanting hepatocellular carcinoid organoids derived from iPSCs into mice to mimic the tumor microenvironment of clinical patients. In vitro gene manipulation of iPSCs, followed by differentiation of the modified iPSCs, resulted in the generation of organoids capable of identifying the role of specific genes in the development of tumors. In 2021, human iPSCs were engineered to carry an inducible H3.3-K27M allele in the endogenous locus, and iPSCs-derived cerebral organoids were established using redifferentiated iPSCs to investigate the effects of the mutation in various diffuse intrinsic pontine glioma (DIPG)-relevant neural cell types [55]. Additionally, organoids produced by iPSCs derived from patients with familial cancer susceptibility syndromes can help researchers comprehend the significance of specific gene mutations in tumor progression. Fibroblasts from familial adenomatous polyposis (FAP) patients were infected with lentiviruses expressing OSKM to generate FAP-iPSCs and FAP-iPSCs, which were then differentiated into colonic organoids (COs) [56]. This method enabled the researchers to investigate the role of genetic factors to the development of colorectal cancer and to screen drugs using COs.

## Application of iPSCs in the study of familial diseases

Pathologically, tumors originate in normal cells that accumulate genetic and epigenetic alterations [57, 58]. However, conventional tumor models usually mimic the advanced stage of tumors and are ineffective at shedding light on the malignant transformation of normal cells. iPSCs derived from somatic cells with germline mutations can help to understand the significance of additional genetic alterations in tumor progression [3] (described in Fig. 3f). Here, we discuss the applications of iPSC technology in understanding hereditary breast and ovarian cancer syndrome (HBOC), Li-Fraumeni syndrome (LFS), and FAP.

In 2013, 24 iPSCs cell lines were derived from fibroblasts of eight individuals from a BRCA1 5382insC mutant family and characterized [59]. Although there was no difference in differentiation ability between BRCA1 wild-type and mutant iPSCs, BRCA1 mutant iPSCs expressed significantly higher protein kinase C-theta (PKC-theta), establishing a correlation between BRCA1 mutation and PKC-theta expression. In 2021, scientists used patient-specific iPSCs to generate induced mesenchymal stem cells (iMSCs) and reported a major effect of BRCA1 haploinsufficiency on the tumor-associated stroma in the context of BRCA1-associated cancers, opening new avenues for personalized treatment and prevention of BRCA1-associated hereditary breast cancer [60].

LFS, caused by germline mutations in the tumor suppressor gene TP53, is associated with the familial occurrence of various cancers [61, 62]. To understand its role in the pathogenesis of osteosarcoma (OS), scientists generated iPSCs from a family of LFS patients [61]. LFS patient-derived iPSCs were then used to establish a human familial cancer model, revealing the role of mutant TP53 in regulating the imprinted gene network, the abnormal regulation of which leads to defective osteoblast differentiation and tumorigenesis. The feasibility of using iPSCs to study hereditary human cancer syndromes was demonstrated. In 2018, LFS patient-derived iPSCs were used to investigate the oncogenic role of secreted frizzled-related protein 2 (SFRP2) in p53 mutation-associated OS development [63].

Using iPSCs derived from normal individuals or FAP patients, Mostoslavsky et al. [64] established a new platform. They provided compelling evidence that APC heterozygosity causes distinct phenotypic and molecular abnormalities that impact basic cellular function and integrity, opening up new perspectives on the earliest stages of APC-mediated tumorigenesis. In 2022, peripheral blood mononuclear cells from a FAP patient carrying a heterozygous one bp deletion in Exon 17 of the APC gene generated iPSCs [65]. The established iPSCs line will facilitate disease modelling of FAP in vitro.

Although iPSCs technology has made some progress studying familial cancers, the cells used for reprogramming are non-tumorigenic normal cells. To fully understand the mechanism, developing iPSCs from different tissues may be necessary to understand why certain tumors are primarily found in specific locations.

## Application of iPSCs in cancer cells reprogramming

In recent years, considerable emphasis has been placed on exploiting the plasticity of cancer cells to reprogram and alter their phenotype. Recent studies have revealed that cancer cell heterogeneity and phenotypic plasticity, which were once considered contributors to invasion and metastasis, can now be exploited to reprogram cancer cells and alter their phenotype. In cell reprogramming, pre-existing chromatin states, DNA methylation, and histone modification must be reset to reactivate the epigenetically silenced regions [66]. Since the successful induction of pluripotent stem cells from mouse somatic cells in 2006, scientists have been experimenting with the applications of transcription factor-mediated reprogramming of cancer cells to reverse the malignant phenotype. Other techniques, including nuclear reprogramming of somatic cells by injection of tumor cell-embryonic carcinomas into normal blastocysts [67], in vitro hybridization of cancer cells with ESCs [68], and somatic cell nuclear transfer (SCNT) technique [69], which implants an enucleated oocyte with a cancer cell nucleus have been used to suppress the tumorigenic phenotype. The reprogramming of tumor cells into a pluripotent state opens new opportunities to explore the potential of normalizing the malignant phenotype of tumor cells in vivo, resulting in PSC-like cells that hold promise as an alternative to conventional cancer treatment. Additionally, this cutting-edge technique may also provide an opportunity to establish larger populations of cancer stem cells (CSCs-like cells), thereby significantly enhancing our ability to investigate the complex biological properties of CSCs. Such research is essential for understanding drug-resistant tumors and laying a solid foundation for developing strategies to reduce cancer recurrence. Various malignant solid tumor cell types reprogrammed into a pluripotent state are summarized in Table 2.

**Table 2:** Overview of reprogramming malignant solid tumor cells into a pluripotent state

Cancer cell type	Reprogramming methods	ES/Pluripotency markers expression	Epigenetic Modification	Teratomas formation	Tumorigenicity	Tumor formation	Drug sensitivity	Differentiation products	Ref
Mouse melanoma cells(R545)	Lentivirus OKM	n.d./ Increased	Demethylation (Oct4 and Nanog promoters)	Yes	Decreased	No tumors in the absence of DOX2	n.a	n.a	[70]
HGC cells	Retrovirus/ lentivirus OSKM	Increased/ n.d	Demethylation (Nanog promoter), histone modification	No	Decreased	Lesser and smaller (postiPC cells)	More sensitive to 5-FU (PotsiPC)	Three germ layers	[72]
Human lung adenocarcinoma cells (A549)	Lentivirus OSLN hypoxia (HIF)	Increased/n.d	Partially unmethylated (OCT4 promoter)	No	Increased	Aggressive tumors	n.a	n.a	[87]
HGC cells (HCT116)	Retrovirus/lentivirus OSKM hypoxia/TP53 null	Increased/n.d	n.d	No	Decreased (hypoxia) Increased (TP53 null)	Lower level (Hypoxia) Higher level (TP53 null)	n.a	Multi-differentiation potential	[73]
Human NSCLC cells (H358, H460)	Retrovirus/lentivirus OSKM	Increased/n.d	Hypomethylation of AMPs	n.a	n.a	n.a	n.a	Three germ layers	[76]
Human sarcoma cells (HOS, SAOS2, MG63, SW872, SKNEP)	Lentivirus OSKMLN	Decreased/ Increased	H3K4triMe- and H3K27triMe-modified (c-Myc promoter), global DNA demethylation	No	Decreased	Slower (rep. cells) No (red. cells)	n.a	Endoderm and ectoderm, mature connective tissue and red blood cells	[78]
Human PDACs	Lentivirus OSKM	n.d./ Increased	Demethylation (Nanogpromoter)	Yes	Increased	PanIN	n.a	Pancreatic intraepithelial neoplasia (PanIN)	[35]
Human GNS cells	Transposon system OK	Increased/Increased	Resetting of DNA methylation, hypermethylated of GFAP	Yes	Decreased	n.d	n.a	Multilineage, but mainly neural lineage	[37]
Human luminal-like breast cancer cells (MCF-7)	Retrovirus OSKM	n.d./ Increased	n.a	No	Increased	More and faster	n.a	n.a	[88]
Human LGGs (BT01,BT03)	Lentivirus OSKM	n.d./ Increased	n.a	Yes	Decreased	No	n.a	Three germ layers	[79]
Human colon cancer cells (LoVo, OUMS-23)	Sendai virus OSKM	n.d./ Increased	n.a	No	Increased	n.d	More resistant to 5-FU	n.a	[89]
Mouse GBM cells (T731, T653)	Retroviral plasmids OSK + PD98059	n.d./ Increased	n.a	n.a	n.a	n.a	n.a	n.a	[165]
Human melanoma cells (A375, MDA-MB-435)	Sendai virus OSKM	n.d./ Increased	n.a	n.a	n.a	n.a	n.a	Three germ layers	[71]
Human PDAC cells (PDAC-247)	Episomal vectors OSKM	n.d./ Increased	Resetting the epigenetic profile	n.a	Decreased	Lesser	n.a	n.a	[74]
Human prostate cancer cells(22RV1)	Sendai virus OSKM	Increased/ n.d	n.a	n.a	n.a	n.a	n.a	Three germ layers	[77]
Human RDEB-cSCCs	Episomal plasmid OSKM	n.d./ Increased	n.a	n.a	Decreased	Slower and smaller (RDEB-cSCC-iKCs)	n.a	Keratinocytes	[75]
Human ovarian cancer cells (PEO4)	Retroviruses OSKM/OSKG	n.d./ Increased	n.a	n.a	n.a	n.a	More resistant to cisplatin and taxol	Three germ layers	[90]
Mouse ESCs-derived germ cell tumors	ESC medium	n.d./ Increased	n.a	Yes	Decreased	n.d	n.a	Embryonic and extraembryonic lineage	[80]

HGC: Human gastrointestinal cancer; NSCLC: non-small cell lung cancer; PDAC: Pancreatic ductal adenocarcinoma; GNS: glioblastoma-derived neural stem cells; LGGs: low grade gliomas; GBM: Glioblastoma multiform; RDEB-cSCCs: Recessive dystrophic epidermolysis bullosa (RDEB)-derived cutaneous squamous cell carcinoma (cSCC); OKM: OCT4, KLF4, c-Myc; OSKM: OCT4, SOX2, KLF4, c-Myc; OSLN: OCT4, SOX2, Lin28, NANOG; OSKMLN: OCT4, SOX2, KLF4, c-Myc, Lin28, NANOG; OK: OCT4, KLF4; OSK: OCT4, SOX2, KLF4; OSKG: OCT4, SOX2, KLF4, GLIS1; n.d.: not determined; n.a.: not applied; rep.: reprogrammed; red.: redifferentiated; PanIN: Pancreatic intraepithelial neoplasia; 5-FU: 5-fluorodeoxyuridine.

## Reprogramming cancer cells into benign cells

Reprogramming cancer cells into iPSCs has emerged as a promising method for resetting the identity of malignant cells without altering the genome sequence of the cell. This technology has prompted researchers to investigate various methods to induce solid tumors into iPSCs and normalize their phenotypes. (described in Fig. 3a, b). Utikal et al. [70] successfully converted mouse melanoma cell lines to a benign phenotype using doxycycline-dependent lentiviruses expressing OCT4, KLF4, and c-Myc. The generated iPSCs exhibited endogenous OCT4, KLF4, and c-Myc expression, demethylation of OCT4 and Nanog promoters, and loss of tumorigenicity in vivo. At five months of age, mouse chimeras derived from the reprogrammed melanoma cells maintained benignity and did not develop visible tumors after the doxycycline-inducible lentiviral expression of Yamanaka factors was terminated. This suggests that the reprogrammed cells underwent a normal differentiation process to produce benign cells in vivo. A detailed protocol for reprogramming human melanoma cell lines A375 and MDA-MB-435 into iPSCs was published in 2019 [71].

Similarly, Yamanaka factors generated iPSCs from gastrointestinal cancer cells, including colorectal, esophageal, gastric, pancreatic, liver, and bile duct cancers [72]. The cancer-derived iPSCs expressed ESCs markers, were more sensitive to differentiation-inducing treatment, exhibited reduced tumorigenicity, and were more sensitive to 5-fluorodeoxyuridine (5-FU) than parental cells. The effects of hypoxia and p53 mutations on reprogramming efficiency were determined in 2012. In hypoxia, wild-type HCT116 cells generated iPSCs at approximately four times higher rates than in normoxia, and TP53 deficiency significantly increased the transformation efficiency of HCT116 cells in normoxia [73].

In 2019, scientists tested three somatic cell reprogramming methods on PDAC primary cancer cultures (PDAC-247). They found that induction with episomal vectors (OCT4, SOX2, KLF4, c-Myc, and LIN28A, combined with P53 knockdown(shP53)) was the most efficient method compared to lentivirus-mediated induction [74]. Reprogrammed cells showed reduced tumorigenicity in vitro and in vitro. Additionally, researchers reprogrammed recessive dystrophic epidermolysis bullosa (RDEB)-derived cutaneous squamous cell carcinoma (cSCC) into RDEB-cSCC-iPSCs by OSKM and then redifferentiated them into keratinocytes (RDEB-cSCC-iKC) [75]. The resultant cells exhibited reduced tumorigenic potential than the parental cells. Furthermore, researchers have successfully induced the transformation of malignant solid tumor cells, including lung cancer [76], prostate cancer [77], sarcoma [78], low-grade gliomas [79], and human germ cell tumors [80], into a pluripotent state using targeted transcription factors. This innovative technique has demonstrated the ability to significantly reduce the tumorigenicity of the original parental cancer cells.

The preceding experiments demonstrate that solid tumor cells are malleable and can be reprogrammed using iPSCs technology to reverse the malignant phenotype of tumors. This discovery has sparked new ideas for treating malignant tumors and encouraged more scientists to use iPSCs technology to develop innovative protocols for cancer treatment. What’s more, iPSCs derived from normal cells have therapeutic effects in addition to reprogramming cancer cells into a pluripotent state to reduce tumorigenicity. In 2019, iPSCs derived from human skin fibroblasts were transplanted into the submandibular gland of rats with salivary gland squamous cell carcinoma [81]. The presence of regenerative tissue in salivary glands treated with iPSCs suggests that iPSCs may be useful in treating salivary gland cancer. The antitumor effect of iPSCs in rat submandibular gland carcinoma was achieved by regulating the apoptotic response and the expression of Sirt 1, TGF β, and MALAT 1 in cancer cells [82].

## Reprogramming cancer cells into CSCs

CSCs, a subset of cells characterized by slow cycling, self-renewal, high tumorigenic potential, and treatment resistance, play a crucial role in the initiation and maintenance of cancer [83]. The presence of CSCs significantly affects the efficacy of treating malignant solid tumors. Patients with acute myeloid leukemia, which can cause hematological malignancies in obese diabetic/severe combined immunodeficient mice, provided the first compelling evidence for the existence of CSCs [84]. Since then, CSCs have been identified in numerous human cancers, and their biological characteristics and significance have been the subject of extensive study [85, 86]. However, little is known about them due to their scarcity, difficulty in isolation, and long-term in vitro culture. Fortunately, iPSCs technology can sever as a platform to investigate CSCs-associated characteristics.

Reprogramming malignant solid tumor cells with iPSCs technology does not always reverse the malignant phenotype; reprogrammed cells may exhibit CSCs-like characteristics. (described in Fig. 3c, d). Reprogramming tumor cells into CSCs and using reprogrammed cells in cancer research will advance our understanding of oncogenic characterization. Transcription factor-mediated iPSCs technology has made significant advancements in CSCs research, including establishing in vitro models of the CSCs-like state and identifying transcriptional regulators associated with CSCs. Hypoxia promotes pluripotency gene expression in various cancer cells, and that reprogrammed lung adenocarcinomas exhibit high tumorigenicity [87]. Reprogramming experiments on MCF-7 human breast cancer using iPSCs technology to establish an in vitro model of the CSCs-like state revealed that transcriptional repression of mTOR blockers is an intrinsic process that occurs during the acquisition of CSCs-like properties in differentiated breast cancer cell populations [88]. This method produced CSCs from colon cancer cell lines LoVo and OUMS-23 [89]. Bindhya et al. [90] reprogrammed the ovarian cancer cell line PEO4 into iPSCs using lentiviral transduction of the Yamanaka factors. Using GLIS1 instead of the c-Myc oncogene and successfully transformed PEO4 cells into iPSCs and demonstrated that PEO4-iPSCs significantly overexpressed CSCs markers, including CD133, EphA1, ALDH1A1, and LGR5, and were more resistant to chemotherapeutic agents. This in vitro model will help to understand the characteristics of CSCs in ovarian carcinogenesis. The iPSCs technology has proven to significantly aid in producing CSCs, thereby significantly advancing our understanding of these cells. However, it is crucial to recognize that not all cancer cells possess the required plasticity to be reprogrammed into a CSCs-like state. Only a small proportion of cancer cell genes can be fully activated to initiate reprogramming. Therefore, it is imperative to focus on generating CSCs from a wider variety of tumors and improving the efficiency of the induction process.

The direct generation of CSCs from cancer cells still faces efficiency challenges, prompting scientists to investigate the potential of utilizing the tumor microenvironment to convert iPSCs into CSCs. Masaharu Seno et al. [91–97] successfully transformed mouse iPSCs into CSCs using a conditioned medium (CM) derived from cancer cells. The transformed cells exhibited stemness, sphere formation ability, differentiation ability, malignant tumorigenicity, and the expression of CSCs markers. In a subsequent study, Masaharu Seno et al. [98] injected a mixture of mouse iPSCs and human pancreatic cancer cells into immunodeficient mice, and the mouse iPSCs were characterized as tumorigenic and self-renewing CSCs. In conclusion, Masaharu Seno's laboratory established a method to transform iPSCs or ESCs into CSCs in the microenvironment. This method employs a CM enriched with growth factors, chemokines, and tissue-specific factors that mimic the microenvironment in which cancer is initiated. This method transformed iPSCs into CSCs with high malignant tumorigenic and metastatic potential. Several additional experiments have shown that iPSCs can be converted into CSCs [99]. The redifferentiation of iPSCs derived from mouse fibroblasts into CSCs lacks specificity, and the replacement of iPSCs derived from cancer cells with those derived from normal cells may result in the generation of CSCs that are more similar to those found in human cancers.

## Application of iPSCs in cancer immunotherapy

**Fig. 4:**
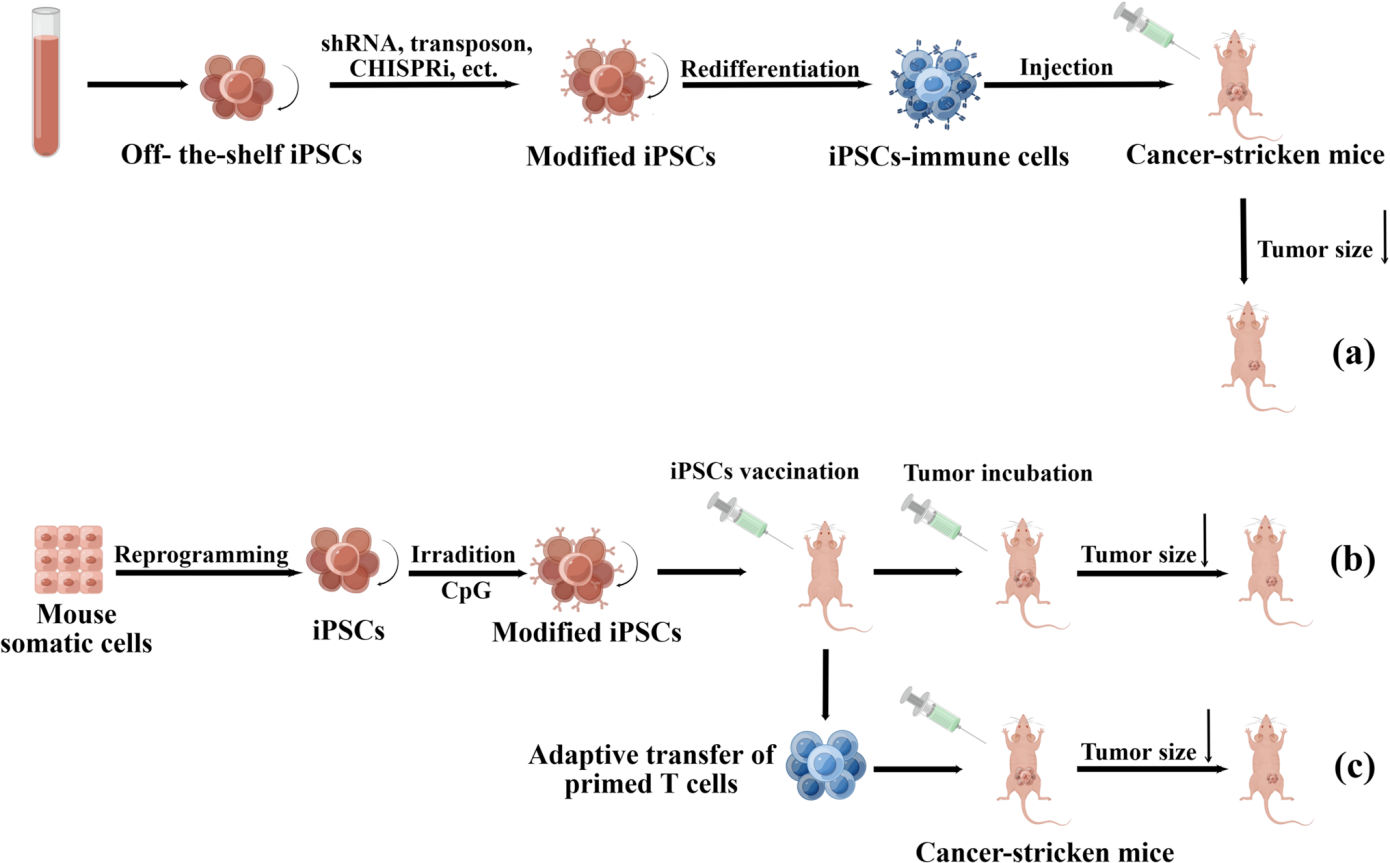
The applications of iPSCs technology in cancer immunotherapy. Off-the-shelf iPSCs can be differentiated into various immune cells after modification, and iPSC-immune cells can reduce the size of tumors (**a**). Modified iPSCs can stimulate anti-tumor responses as cancer vaccines because they share cellular and molecular characteristics with some cancer cells (**b**). T cells from vaccinated mice can also stimulate anti-tumor responses (**c**)

Adoptive cell therapy (ACT), a major form of immunotherapy, involves infusing tumor-infiltrating lymphocytes or peripheral blood-derived immune cells into patients and has shown efficacy in treating malignant tumors [100]. However, clinical trials using these primary immune cells have shown their limitations, including cytokine release syndrome, cytopenias, neurologic events, and febrile neutropenia, accompanied by cytotoxicity against B cell malignancies when infusing autologous peripheral blood (PB)-derived CAR-T cells into patients [101]. Although ESCs are pluripotent cells capable of differentiating into most immune system cells and can be used to produce cancer vaccines [102–104], the clinical applications of ESCs are restricted due to their limited source and the associated ethical concerns. iPSCs technology may resolve these issues by developing cancer vaccines for specialized cancer treatment and specialized immune cells with anti-cancer potential [105]. The applications of iPSCs technology in cancer immunotherapy are described in Fig. 4.

## iPSCs-derived T cells

Most immunotherapies aim to stimulate cytotoxic T cells (CTLs) that target tumors to cure patients [106]. However, the short lifespan of activated CTLs often limits the efficacy of such regimens due to antigen-induced cell death. The applications of iPSCs technology to clone and expand tumor antigen-specific T cells may be able to address this issue. Using Yamanaka factors, scientists established iPSCs from mature cytotoxic T cells specific for the melanoma epitope MART-1 in 2013. They redifferentiated them into T cells, resulting in the expansion of antigen-specific T cells [107]. However, the potential of these cells to treat tumors was not demonstrated. In 2018, scientists reported that CD8ab T cells derived from antigen-specific T cells (T-iPSCs) derived from human iPSCs lose their antigen specificity [108]. CRISPR knockout of a recombinase gene in the T-iPSCs prevented this additional T cell receptor (TCR) rearrangement, resulting in the generation of antigen-specific T cell receptor-stabilized CD8Ab T cells that effectively inhibited the growth of ovarian cancer KOC7C cell line and hepatocellular carcinoma HepG2 cell line in xenograft tumor models. In 2022, researchers developed developmentally mature chimeric antigen receptor (CAR) T cells from iPSCs by combining a stroma-free differentiation system with EZH1-repression-mediated epigenetic reprogramming [109]. These CAR T cells demonstrated enhanced anti-tumor activities and suppressed tumor growth in vitro and in an immunodeficient non-obese diabetic-SCID IL2Rgammanull (NSG) mice model with lymphoma cells. Additionally, scientists established a universal iPSCs source for allogeneic T-cell therapy by knocking out HLA-I- and HLA-II-related genes and a NK cell-activating ligand gene and transducing the NK-inhibitory ligand-scHLA-E in an iPSCs clone [110]. The hypoimmunogenic T cells derived from iPSCs may contribute to developing off-the-shelf T cell immunotherapies for cancers. Several additional studies have reported the enhanced anti-tumor activity of modified iPSCs-T cells [111–115].

## iPSCs-derived NK cell

Natural killer (NK) cells are a type of intrinsic lymphoid-like cells that can kill tumor target cells by upregulating regulatory receptors, antibody-dependent cell-mediated cytotoxicity (ADCC) effects, and expressing cytokine receptors [116]. However, the large-scale isolation of NK cells from donors is difficult. Recent efforts have been made to generate NK cells through targeted differentiation of iPSCs, which paves the way for the application of cell reprogramming in immunotherapy [117]. In contrast to primary NK cells, those derived from iPSCs can be prepared with homogeneous quality and modified easily to exert a desired response against tumor cells [118]. In 2018, NK cells were generated from human iPSCs that express a particular CAR called NK-CAR-iPSC-NK cells, which exhibited improved anti-tumor activity [119]. In 2020, Cichocki et al. [120] induced iPSCs into NKs (iNKs) using a cocktail of small molecules and cytokines, which can produce inflammatory cytokines and exert potent cytotoxicity against various hematological and solid tumors. Cichocki et al. triple-modified iPSC-derived NK cells (iDuo NK cells) to express a CD19 targeting CAR for antigen specificity, a high affinity, non-cleavable CD16 (hnCD16) to enhance innate ADCC, and a membrane-bound IL-15/IL-15R fusion protein (IL-15RF) for enhanced persistence. In multiple in vitro and xenogeneic adoptive transfer models, iDuo NK cells demonstrated robust anti-lymphoma activity [121]. Several comparable studies have reported the feasibility of redifferentiating iPSCs into NK cells with anti-tumor effects [122–125].

## iPSCs-derived other immune cells

Additionally, macrophages [126–128], mucosal-associated invariant T (MAIT) cells [129], invariant natural killer T (iNKT) cells [130, 131], myelomonocytic cells [132], cytotoxic γδ natural killer T (NKT) cells [133], and dendritic cells (DC) [134] can also be derived from iPSCs for the treatment of malignant solid tumors. The original anti-cancer function of immune cells was maintained. The use of iPSCs technology has facilitated the generation of numerous immune cells, offering a partial solution to the challenge of isolating and expanding immune cells from autologous sources. In addition, anti-tumor clinical trials with iPSCs-derived platelets [135, 136] and iPSCs-derived NK cells have been conducted [125, 137, 138]. These cells provide standardized, targeted “off the shelf” lymphocytes for immunotherapy against cancer.

## iPSCs meet cancer vaccine

Cancer cells and embryonic tissues share cellular and molecular characteristics, suggesting that iPSCs could be used as cancer vaccines to stimulate anti-tumor responses. Schöne first proposed vaccinating animals with fetal tissue to prevent the growth of transplantable tumors over a century ago [139]. Subsequent studies revealed a significant gene expression and antigens overlap of the human iPSCs- and human ESCs-derivatives with various cancers [140, 141]. Based on these findings, researchers hypothesized that ESCs and iPSCs could be used to stimulate antitumor responses. In 2009, scientists discovered that mice immunized with hESCs line H9 generated consistent cellular and humoral immune responses against CT26 colon carcinoma [103]. Later, scientists evaluated a similar approach using xenogeneic human ESCs as a preventive cancer vaccine in a rat ovarian cancer model and observed a tumor-prevention effect [142]. Although hESC-based vaccination is a promising immunotherapy modality for ovarian cancer, ethical issues and limited sources pose challenges for ESCs-related research. Embryonic/fetal material or ESCs are often derived from unrelated donors and may express mismatched MHCs, stimulating an immune response. Additionally, the tumorigenicity of ESCs has been a significant obstacle to their clinical use as cancer vaccines [143]. Studies have shown that radiation can significantly reduce the tumorigenicity of iPSCs [144], and it has been hypothesized that undifferentiated iPSCs are immunogenic and could be used as cancer vaccines [145].

In a transplantable mouse colon cancer model, scientists evaluated the efficacy of human iPSCs line TZ 1 as an anticancer vaccine [103]. These iPSCs induced high IFNγ and IL-4 production in splenocytes against mouse colon cancer cells. In 2018, researchers improved the iPSCs vaccine by adding the immunostimulatory adjuvant CpG oligodeoxynucleotide (C + I Vaccination) and irradiating iPSCs to prevent teratoma formation in mice [146]. Mice that received the iPSCs-based cancer vaccine could reject transplanted breast cancer, melanoma, and mesothelioma cells. In addition, it was shown that the C + I vaccine provides breast cancer and melanoma immunity by enhancing antigen presentation and T-helper/cytotoxic T-cell activity (described in Fig. 4b, c). In 2021, the C + I vaccine was proven to stimulate cytotoxic anti-tumor CD8 T cell effector and memory responses, induce cancer-specific humoral immune responses, reduce immunosuppressive CD4 T regulatory cells, and prevent tumor formation in 75% of PDAC mice [147]. Using iPSCs, gene editing, and tumor-targeted replicating oncolytic viruses, scientists developed a novel individualized prophylactic and therapeutic vaccination regimen for pancreatic cancer [148]. In addition, several other studies have also utilized iPSCs technology to develop vaccines against tumor growth and metastasis [102, 149, 150]. However, the vaccines mentioned earlier are primarily prophylactic, and their significance for pre-existing tumors is limited. The use of iPSCs technology to develop therapeutic vaccine development has the potential to facilitate the treatment of existing cancers.

## Challenges and future directions

Reprogramming somatic and cancer cells to iPSCs is no longer a technical challenge. However, limited data are available on the applications of iPSCs technology to malignant solid tumors and are accompanied by low efficiency, tumorigenicity, immunogenicity, and genetic abnormalities. Utikal et al. [70] reported an efficiency of only 0.05%-0.1% in reprogramming malignant melanoma. Using autologous cells is costly and time-consuming, and some patients cannot wait too long for treatment, or other treatments may alter the characteristics of the tumor.

While infinite proliferation is advantageous for regenerative purposes, it can become a double-edged sword if cells continue to proliferate uncontrollably even after transplantation, resulting in tumor formation. Additionally, reprogramming factors used in producing iPSCs have been found to exhibit tumorigenic capabilities, resulting in the activation of some oncogenes during the induction of pluripotency [151–158]. In some cases, deleting specific oncogenes may improve reprogramming efficiency while increasing tumorigenicity. The epigenetic reactivation of pluripotent genes can contribute to the activation of oncogenes, making it difficult to ensure the safety of iPSCs-based therapies.

The use of allogeneic-derived iPSCs raises immunogenicity concerns. Although allogeneic-derived iPSCs were initially believed to be immunotolerant, Zhao et al. [159] first reported immune rejection in autologously transplanted mouse iPSCs. Scientists have investigated numerous methods for reducing the immunogenicity of iPSCs [160]. Additionally, the generation of iPSCs can result in unpredictable genetic changes and such aneuploidy may limit the differentiation capacity, raise safety concerns for therapeutic applications, and increase the risk of tumorigenicity [161, 162].

In the realm of iPSCs technology, multiple issues still require attention. Numerous efforts have been made to improve the efficiency and security of iPSCs technology. Recent advancements in single-cell RNA sequencing have provided researchers with a more comprehensive understanding of various cancer cells. Many transcription factor candidates also show great promise for enhancing cancer cell reprogramming efficiency.

Additionally, direct reprogramming offers the enticing possibility of bypassing the pluripotent stem cell stage, thereby reducing the risk of inducing malignant transformation in pluripotent cancer cells and partially addressing concerns about efficacy and safety. The successful application of small molecule cocktails in somatic cell reprogramming possesses significant potential as a chemical induction method for reprogramming malignant solid tumor cells. The development of iPSCs banks has highly facilitated the applications of iPSCs by offering readily available off-the-shelf products.

## Conclusion

The iPSCs technology has emerged as a valuable tool for disease modeling and treating malignant solid tumors. While certain challenges remain, ongoing advancements in cell manufacturing and genome editing technologies are anticipated to pave the way for further exploration of iPSCs applications in treating malignant solid tumors. As these obstacles are gradually eliminated, the potential for more patients to benefit from clinical cancer treatments utilizing iPSCs technology becomes increasingly promising.

## Data Availability

Not applicable.

## Abbreviations

*iPSCs*: Induced pluripotent stem cells
*ESCs*: Embryonic stem cells
*CiPSCs*: Chemically induced pluripotent stem cells
*OSKM*: OCT4, SOX2, KLF4, c-Myc
*OSLN*: OCT4, SOX2, Lin28, NANOG
*OSKMLN*: OCT4, SOX2, KLF4, c-Myc, Lin28, NANOG
*OKM*: OCT4, KLF4, c-Myc
*OK*: OCT4, KLF4
*OSK*: OCT4, SOX2, KLF4
*OSKG*: OCT4, SOX2, KLF4, GLIS1
*PDX*: Patient-derived xenografts
*PDAC*: Pancreatic ductal adenocarcinoma
*GNS*: Glioblastoma-derived neural stem cells
*PDO*: Patient-derived organoids
*HCC*: Human hepatocellular carcinoma
*FAP*: Familial adenomatous polyposis
*HBOC*: Hereditary breast and ovarian cancer syndrome
*LFS*: Li-Fraumeni syndrome
*5-FU*: 5-Fluorodeoxyuridine
*CSCs*: Cancer stem cells
*CAR*: Chimeric antigen receptor
*PSC*: Pluripotent stem cell
*NK*: Natural killer
*HiPSCs*: : Human-induced pluripotent stem cell
*n.d.*: Not determined
*n.a.*: Not applied
*rep.*: Reprogrammed
*red.*: Redifferentiated
*HGC*: Human gastrointestinal cancer
*NSCLC*: Non-small cell lung cancer
*PanIN*: Pancreatic intraepithelial neoplasia
*RDEB-cSCCs*: Recessive dystrophic epidermolysis bullosa (RDEB)-derived cutaneous squamous cell carcinoma (cSCC)
*GBM*: Glioblastoma multiform
*LGGs*: Low grade gliomas
